# The lactate to albumin ratio linked to all-cause mortality in critically ill patients with septic myocardial injury

**DOI:** 10.3389/fcvm.2023.1233147

**Published:** 2023-09-13

**Authors:** Sheng Chen, Senhong Guan, Zhaohan Yan, Fengshan Ouyang, Shuhuan Li, Lanyuan Liu, Jiankai Zhong

**Affiliations:** ^1^ Department of Cardiology, Shunde Hospital, Southern Medical University, Foshan, China; ^2^ Department of Rehabilitation Medicine, Shunde Hospital, Southern Medical University, Foshan, China; ^3^ Department of Pediatrics, Shunde Hospital, Southern Medical University, Foshan, China; ^4^ Department of Ultrasound Medicine, Shunde Hospital, Southern Medical University, Foshan, China

**Keywords:** lactate/albumin ratio, septic myocardial injury, cox proportional hazards regression, intensive care unit, all-cause mortality

## Abstract

**Background:**

The lactate to albumin ratio (LAR) has emerged as a promising prognostic marker in critically ill patients. Despite its potential utility, the prognostic value of LAR in septic myocardial injury (SMI) remains uncertain.

**Methods:**

This study aims to investigate the prognostic significance of LAR in SMI through a retrospective cohort analysis of data from the Medical Information Mart for Intensive Care III (MIMIC-III) (v1.4) database. The study included intensive care unit (ICU)-admitted patients (age ≥18 years) diagnosed with SMI. The primary endpoint was in-hospital mortality.

**Results:**

A total of 704 patients were included in the study, of which 59.10% were male. Hospital mortality and ICU mortality rates were recorded at 29.97% and 22.87%, respectively. After adjusting for confounding factors, multivariate Cox proportional risk analysis demonstrated that LAR was independently associated with an increased risk of both hospital mortality (HR, 1.39 [95% CI: 1.24–1.56] *P* < 0.001) and ICU mortality (HR, 1.46 [95% CI: 1.29–1.65] *P* < 0.001). Furthermore, the generalized additive model (GAM) and restricted cubic spline (RCS) model indicated a linear relationship between LAR and mortality rates in the ICU and hospital.

**Conclusions:**

The LAR may serve as a potential prognostic biomarker in critically ill patients with SMI. High LAR levels are associated with a higher risk of in-hospital mortality and can help identify individuals with high mortality rates. Overall, the findings emphasize the importance of using LAR as a tool for risk stratification and management of critically ill patients with SMI.

## 1. Introduction

Sepsis, a life-threatening condition caused by an overwhelming immune response to severe infection, is a significant contributor to global mortality. It has been estimated that approximately 30 million individuals are affected by sepsis annually, and the associated mortality rate is alarmingly high with 6 million deaths annually (1, 2). Multi-organ dysfunction is a common complication of sepsis and myocardial injury is frequently observed in sepsis patients, leading to myocardial dysfunction in more than 50% of patients (3, 4). Sepsis with myocardial injury is defined as SMI, which can lead to complications such as severe arrhythmias, heart failure and cardiogenic shock (5). The early management of SMI plays a crucial role in improving patient prognosis. Therefore, it is imperative to establish a hierarchical management approach for SMI, which involves intervening based on risk stratification. Patients with a low-risk stratification should be closely observed, while those with a high-risk stratification require active intervention to effectively enhance patient prognosis and possibly reduce SMI mortality rates. Given these factors, there is an urgent requirement in clinical practice to identify easily obtainable and sensitive indicators for predicting the prognosis of SMI.

Elevated levels of blood lactate, which are indicative of tissue and organ dysfunction and low tissue perfusion, can result from anaerobic metabolism (6, 7). Meanwhile, albumin is a negative acute phase protein whose levels exhibit a negative correlation with the degree of inflammation (8–10). Prior studies have identified a relationship between sepsis status and prognosis and arterial blood lactate or serum albumin concentration (10–14). However, measures of lactate and albumin may be subject to some limitations as they can be influenced by other factors (15–17). Studies have suggested that the combination of these two measures in the form of LAR may provide more accurate predictive ability for sepsis compared to lactate alone (18, 19). Moreover, recent research has found that LAR is a reliable predictor of mortality in patients with heart failure and myocardial infarction (20, 21). Although one study (Zhang et al., *n* = 72) has evaluated the diagnostic value of LAR in severe pneumonia and myocardial injury, its prognostic value remains unexplored (22). Accordingly, this retrospective study aims to investigate the association between LAR and prognosis in patients suffering from SMI. Specifically, we analyze the value of LAR in assessing the status and predicting the prognosis of SMI.

## 2. Participants and methods

### 2.1. Data source

This was a retrospective observational study. The data for this analysis were obtained from the MIMIC-III database (v1.4). MIMIC-III is a large, open-access database containing information on patients admitted to ICU at a large tertiary hospital in Boston from 2001 to 2012 (23). In order to access this database, the first author of this study, Sheng Chen, completed the Collaborative Institutional Training Initiative (CITI) course and passed both the “Conflicts of Interest” and “Data or Specimens Only Research” exams (ID: 12046100). The database was approved for research use by Massachusetts Institute of Technology and Beth Israel Deaconess Medical Center review boards, and informed consent was waived.

### 2.2. Population selection criteria

**Figure 1:**
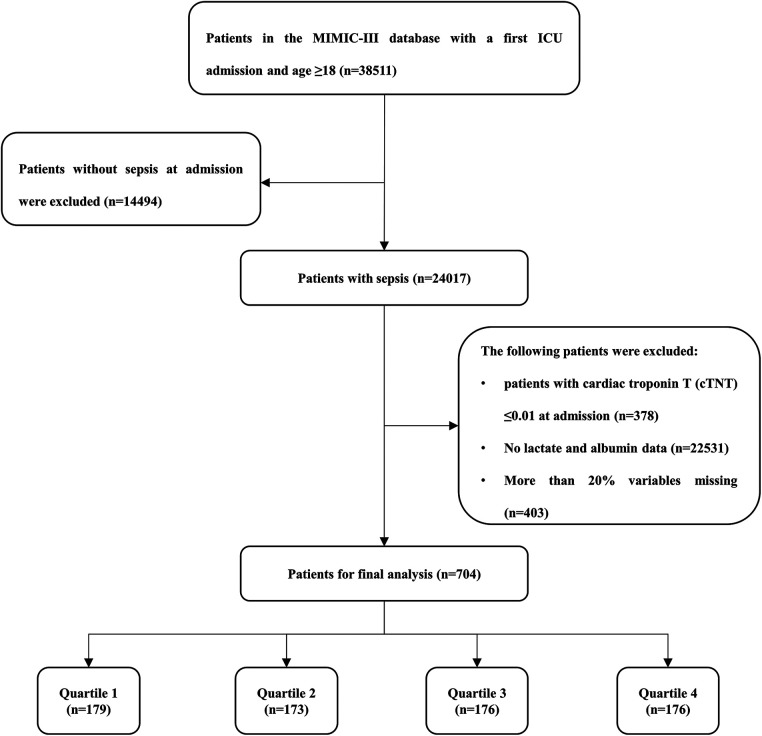
Study flow diagram in the present study.

According to the definition of sepsis 3.0 (2), adult patients (≥18 years) with sepsis who had been hospitalized in the ICU at first admission were included. Patients with the following criteria were excluded: (1) patients with cardiac troponin T (cTNT) ≤0.01 ng/ml at admission were excluded; (2) no lactate or albumin data during the ICU stay; (3) more than 20% variables missing. Finally, a total of 704 patients were included in the study cohort, and were divided into four groups according to the LAR quartile of the first day of hospitalization in ICU (Figure 1).

### 2.3. Variables

We use the Structured Query Language (SQL) with PostgreSQL (version 15.2) to extract data. The baseline characteristics included age, gender and weight. The comorbidities were defined with ICD-9 codes, including coronary artery disease (CAD), heart failure, hypertension, diabetes, hyperlipidemia, chronic kidney disease (CKD) and stroke. The following laboratory variables within the first day after ICU admission have also been extracted, including white blood cell (WBC), neutrophil percentage, lymphocyte percentage, platelet, hemoglobin, hematocrit, potassium, sodium, albumin, lactate, serum creatinine (Scr), blood urea nitrogen (BUN), cTNT, creatine kinase MB isoenzyme (CK-MB), alanine aminotransferase (ALT), aspartate aminotransferase (AST). The LAR was defined as the ratio of the lactate level to the albumin level. It was calculated as lactate (mmol/L)/[albumin (g/dl) × 10]. Furthermore, we also extracted the relevant severity scores for sepsis, including Sequential Organ Failure Assessment (SOFA) score, Systemic inflammatory response syndrome (SIRS) score, quick Sequential Organ Failure Assessment (qSOFA) score, Acute physiology score III (APS III), Simplified acute physiological score II (SAPS II). Finally, we also extracted the clinical treatment, including vasopressin use, ventilator use, and renal replacement therapy (RRT). The participants were followed from the date of admission to the date of death, or until hospital discharge.

To avoid bias, variables with more than 20% missing are excluded, such as C-reactive protein, lactate dehydrogenase, and height. A single imputation method was used to impute missing values for variables with up to 5% missing. For variables with missing values greater than 5% and less than 20%, we use the multiple imputation method and select the relatively optimal data set to impute the missing values (Supplementary Table S1).

### 2.4. Endpoint events and clinical definition

The primary study endpoint was in-hospital all-cause mortality, including hospital mortality and ICU mortality. According to the definition of sepsis3.0, the diagnostic criteria for sepsis is infection + SOFA score ≥2 points. We defined sepsis patients with cTnT levels above the upper limit of normal reference values (cTnT >0.01 ng/ml) as SMI, and excluded acute coronary syndrome (ACS).

### 2.5. Statistical analysis

Student's *t*-test or Kruskal–Wallis *H*-test were used to compare continuous variables, which are presented as mean ± standard deviation or median with interquartile range. Categorical variables are presented as frequencies and percentages, and differences between groups were compared using Pearson chi-square test or Fisher's exact test. We used Kaplan-Meier survival analysis to assess the incidence of primary outcome events between groups according to different levels of LAR, and differences between groups were assessed using log-rank tests. The hazard ratio (HR) and 95% confidence interval (CI) between LAR and primary endpoints were estimated using Cox proportional hazard models and adjusted for multiple models. We also calculated the variance inflation factor to avoid overfitting the model due to multicollinearity between variables. Variables with a variance inflation factor ≥5 were excluded. Based on the inclusion of LAR groups according to quartiles, four multivariate models were constructed. Model 1: unadjusted; Model 2: adjusted for age, gender, and weight; Model 3: adjusted for age, gender, weight, CAD, heart failure, atrial fibrillation, hypertension, diabetes, hyperlipidaemia, CKD, stroke, vasopressin use, ventilator use, and RRT; Model 4: adjusted for age, gender, weight, CAD, heart failure, atrial fibrillation, hypertension, diabetes, hyperlipidaemia, CKD, stroke, vasopressin use, ventilator use, RRT, WBC, neutrophil percentage, lymphocyte percentage, platelets, neutrophil/lymphocyte ratio (NLR), platelet/neutrophil ratio (PNR), platelet/lymphocyte ratio (PLR), hemoglobin, potassium, sodium, Scr, BUN, CK-MB, ALT, AST, and SIRS score. We used RCS model and GAM to examine the associations between LAR and outcomes. Based on the above results, we selected the lowest quartile of LAR values as the reference group. To determine the consistency of the prognostic value of LAR for the primary outcomes, we further stratified the analyses by age (<65 and ≥65 years), gender, CAD, heart failure, atrial fibrillation, hypertension, diabetes, hyperlipidemia, CKD, stroke, vasopressin use, ventilator use, and RRT. Likelihood ratio tests were used to examine interactions between the LAR and the variables used for stratification. The predictive ability of the LAR, lactate and albumin for in-hospital mortality was assessed using receiver operating characteristic (ROC) curves. Integrated discrimination improvement (IDI) and net reclassification improvement (NRI) are used to estimate the improvement in predictive ability after adding LAR to the scoring system. All statistical analyses were conducted with SPSS 27.0 software (IBM SPSS Statistics, Armonk, NY, USA) and R software version 4.2.2 (Institute for Statistics and Mathematics, Vienna, Austria). Statistical significance was defined as a two-tailed *P*-value <0.05.

## 3. Results

After reviewing the data of 38,511 patients who were admitted into the ICU from the MIMIC-III database, a total of 704 patients were included in this study. There were 288 (40.90%) females and 416 (59.10%) males. The median age of the enrolled patients was 68.94 years. The median LAR for all patients was 0.07. The hospital- and ICU-related mortality rates were 29.97% and 22.87%, respectively (Table 1).

**Table 1:** Baseline characteristics of participants according to the LAR quartiles.

Characteristics	Overall (*n* = 704)	Q1 (*n* = 179)	Q2 (*n* = 173)	Q3 (*n* = 176)	Q4 (*n* = 176)	*P*-value
Age, years	68.94 (57.78, 80.55)	69.87 (59.77, 79.83)	69.21 (60.86, 80.07)	73.05 (59.60, 81.94)	64.41 (52.05, 79.32)	0.021
Gender, *n* (%)						0.698
Female	288 (40.90)	68 (37.99)	73 (42.20)	77 (43.75)	70 (39.77)	
Male	416 (59.10)	111 (62.01)	100 (57.80)	99 (56.25)	106 (60.23)	
Weight, Kg	80.00 (67.00, 96.67)	80.00 (68.00, 94.50)	80.00 (68.25, 98.30)	78.50 (65.73, 93.17)	81.80 (62.40, 100.00)	0.612
Comorbidities, *n* (%)						
CAD	223 (31.70)	67 (37.43)	62 (35.84)	58 (32.95)	36 (20.45)	0.002
Heart failure	306 (43.50)	89 (49.72)	83 (48.00)	77 (43.75)	57 (32.38)	0.004
Atrial fibrillation	262 (37.22)	74 (41.34)	70 (40.46)	66 (37.50)	52 (29.55)	0.089
Hypertension	306 (43.50)	76 (42.46)	79 (45.66)	74 (42.05)	77 (43.75)	0.904
Diabetes	266 (37.80)	72 (40.22)	61 (35.26)	77 (43.75)	56 (31.82)	0.100
Hyperlipidemia	196 (27.84)	56 (31.28)	46 (26.59)	49 (27.84)	45 (25.57)	0.649
CKD	218 (30.96)	67 (37.43)	57 (32.95)	52 (29.55)	42 (23.86)	0.043
Stroke	57 (8.09)	15 (8.38)	18 (10.40)	14 (7.95)	10 (5.68)	0.450
Laboratory parameters						
WBC, K/ul	12.70 (8.60, 17.60)	11.60 (8.40, 15.70)	13.50 (8.95, 17.60)	13.95 (9.83, 19.90)	11.95 (7.33, 18.05)	0.004
Neutrophil, %	83.95 (76.00, 88.97)	83.90 (77.00, 89.00)	84.20 (77.25, 88.75)	84.70 (76.55, 89.00)	83.00 (68.35, 88.25)	0.034
Lymphocyte, %	8.30 (5.10, 13.00)	9.10 (5.10, 13.40)	7.90 (5.30, 13.50)	7.20 (4.43, 11.07)	9.15 (5.63, 13.30)	0.050
Platelet, K/ul	203.00 (140.25, 274.00)	220.00 (165.00, 271.00)	192.00 (146.00, 258.00)	215.00 (140.00, 301.00)	167.50 (102.00, 265.25)	<0.001
NLR	9.94 (5.98, 16.65)	9.01 (6.09, 17.06)	10.47 (5.67, 16.64)	11.48 (7.04, 19.91)	8.52 (5.82, 14.83)	0.035
PNR	20.57 (12.91, 31.21)	23.31 (15.72, 36.94)	19.93 (11.89, 28.27)	20.08 (12.69, 27.93)	17.97 (11.67, 32.49)	0.004
PLR	195.16 (119.89, 337.87)	229.16 (138.98, 395.17)	169.32 (117.04, 307.54)	209.36 (129.54, 403.12)	168.21 (101.07, 266.64)	<0.001
Hemoglobin, g/dl	10.70 (9.20, 12.30)	10.30 (9.00, 11.90)	10.90 (9.55, 12.70)	10.60 (9.03, 12.40)	10.50 (9.13, 12.10)	0.130
Hematocrit, %	32.20 (27.80, 37.00)	31.30 (27.40, 35.50)	33.30 (28.80, 37.60)	32.60 (27.80, 37.75)	31.65 (27.33, 37.35)	0.103
Potassium, mEq/L	4.20 (3.70, 4.70)	4.10 (3.70, 4.60)	4.20 (3.80, 4.80)	4.20 (3.70, 4.70)	4.20 (3.80, 4.90)	0.408
Sodium, mEq/L	139.00 (136.00, 142.00)	139.00 (135.00, 142.00)	139.00 (136.00, 141.00)	139.00 (136.00, 142.75)	140.00 (136.00, 143.00)	0.154
Lactate, mmol/L	2.10 (1.40, 3.60)	1.10 (0.90, 1.30)	1.80 (1.55, 2.10)	2.60 (2.13, 3.28)	5.15 (4.00, 7.47)	<0.001
Albumin, g/dl	3.00 (2.50, 3.40)	3.20 (2.90, 3.60)	3.10 (2.70, 3.40)	2.90 (2.40, 3.30)	2.60 (2.20, 3.00)	<0.001
Scr, mg/dl	1.50 (1.00, 2.30)	1.40 (0.90, 2.60)	1.50 (1.10, 2.10)	1.40 (1.00, 2.20)	1.70 (1.10, 2.60)	0.127
BUN, mg/dl	31.00 (20.00, 50.75)	31.00 (19.00, 54.00)	33.00 (19.50, 51.00)	30.00 (20.00, 48.25)	32.00 (20.00, 49.75)	0.795
cTNT, ng/ml	0.11 (0.04, 0.36)	0.09 (0.04, 0.34)	0.10 (0.04, 0.39)	0.12 (0.04, 0.32)	0.12 (0.05, 0.48)	0.861
CK-MB, ng/ml	9.00 (5.00, 22.00)	7.00 (4.00, 15.00)	8.20 (4.30, 22.00)	9.00 (5.00, 17.00)	13.30 (7.00, 35.00)	<0.001
ALT, IU/L	47.00 (24.00, 106.00)	31.00 (19.00, 70.35)	42.00 (21.50, 90.23)	46.00 (27.25, 91.98)	83.05 (40.00, 290.25)	<0.001
AST, IU/L	70.28 (37.00, 174.75)	52.00 (27.00, 104.00)	65.00 (38.50, 119.00)	64.53 (37.25, 137.94)	156.50 (59.75, 474.50)	<0.001
LAR	0.07 (0.05, 0.12)	0.03 (0.03, 0.04)	0.06 (0.05, 0.07)	0.09 (0.08, 0.12)	0.19 (0.15, 0.28)	<0.001
Scoring systems						
SOFA score	7.00 (5.00, 10.00)	6.00 (5.00, 8.00)	7.00 (5.00, 9.50)	7.00 (5.00, 10.00)	10.00 (7.00, 13.00)	<0.001
SIRS score	3.00 (3.00, 4.00)	3.00 (3.00, 4.00)	3.00 (3.00, 4.00)	3.50 (3.00, 4.00)	4.00 (3.00, 4.00)	<0.001
qSOFA score	2.00 (2.00, 3.00)	2.00 (2.00, 3.00)	2.00 (2.00, 3.00)	2.00 (2.00, 2.75)	2.00 (2.00, 3.00)	0.355
APSⅢ	64.00 (49.00, 82.00)	53.00 (40.00, 73.00)	60.00 (46.00, 74.50)	66.00 (52.25, 81.00)	78 (62.25, 98.00)	<0.001
SAPS II	49.00 (39.00, 60.00)	45.00 (37.00, 53.00)	47.00 (37.00, 58.00)	49.00 (39.00, 60.00)	55.50 (44.00, 66.00)	<0.001
Clinical treatment						
Vasopressin use, *n* (%)	283 (40.19)	49 (27.37)	63 (36.42)	72 (40.91)	99 (56.25)	<0.001
Ventilator use, *n* (%)	505 (71.73)	125 (69.83)	110 (63.58)	122 (69.32)	148 (84.09)	<0.001
RRT, *n* (%)	131 (18.61)	33 (18.44)	24 (13.87)	27 (15.34)	47 (26.70)	0.010

LAR, lactate to albumin ratio; CAD, coronary artery disease; CKD, chronic kidney disease; WBC, white blood cell; NLR, neutrophil/lymphocyte ratio; PNR, platelet/neutrophil ratio; PLR, platelet/lymphocyte ratio; SCr, serum creatinine; BUN, blood urea nitrogen; cTNT, cardiac troponin T; CK-MB, creatine kinase MB isoenzyme; ALT, alanine aminotransferase; AST, aspartate aminotransferase; SOFA, Sequential Organ Failure Assessment; SIRS, Systemic inflammatory response syndrome; qSOFA, quick Sequential Organ Failure Assessment; APSIII, Acute physiology score III; SAPSII Simplifed acute physiological score II; RRT, renal replacement therapy.

### 3.1. Baseline characteristics

Table 1 shows the baseline characteristics of the study patients according to LAR quartiles. Patients were categorized according to the LAR values on admission (quartile [Q] 1: 0.02–0.05; Q2: 0.05–0.07; Q3: 0.07–0.12; Q4: 0.12–1.13). The median values of LAR of the four groups were 0.03, 0.06, 0.09 and 0.19, respectively. Patients with higher LAR had higher admission sickness scores, higher rates of vasopressin use, ventilation and RRT, higher WBC, lactate, albumin, CK-MB, ALT, AST and lower platelets, PNR, PLR compared with the lower group. Table 2 shows the outcome events of the participants according to LAR quartiles. With increasing LAR value, ICU mortality (13.41% vs. 17.92% vs. 22.16% vs. 38.07%, *P* < 0.001) and hospital mortality (19.55% vs. 25.43% vs. 31.25% vs. 43.75%, *P* < 0.001) gradually increased.

**Table 2:** The outcome events of participants according to the LAR quartiles.

Events	Overall (*n* = 704)	Q1 (*n* = 179)	Q2 (*n* = 173)	Q3 (*n* = 176)	Q4 (*n* = 176)	*P*-value
LOS ICU, days	5.63 (2.76, 12.21)	4.44 (2.48, 10.21)	5.74 (2.96, 11.36)	4.90 (2.63, 12.99)	6.64 (2.67, 14.15)	0.348
LOS Hospital, days	11.90 (6.60, 21.68)	10.59 (6.19, 19.15)	11.82 (7.68, 20.62)	13.55 (6.08, 21.38)	12.86 (5.22, 26.14)	0.488
ICU mortality, *n* (%)	161 (22.87)	24 (13.41)	31 (17.92)	39 (22.16)	67 (38.07)	<0.001
Hospital mortality, *n* (%)	211 (29.97)	35 (19.55)	44 (25.43)	55 (31.25)	77 (43.75)	<0.001

LOS, length of stay; ICU, intensive care unit.

The baseline characteristics of the survivor and non-survivor groups are summarized in Supplementary Table S2. Patients in the non-survivor group were older and had a lower prevalence of CAD, heart failure and CKD (*P* < 0.05). Regarding of laboratory parameters, participants with an endpoint event had higher levels of potassium, lactate, BUN, CK-MB, ALT, and AST, but lower levels of lymphocyte percentage, platelets, PNR, and albumin (*P* < 0.05). No significant differences were observed in gender, weight, hypertension, diabetes, hyperlipidemia, stroke, WBC, neutrophil percentage, NLR, PLR, hemoglobin, hematocrit, sodium, Scr, and cTNT (*P* > 0.05). SOFA scores, SIRS scores, APS III and SAPS II were higher in the non-survivor group than in the survivor group. In the non-survivor group, the level of LAR was significantly higher than that of the survivor group (0.09 vs. 0.07, *P* < 0.001).

### 3.2. LAR level and in-hospital mortality

**Figure 2:**
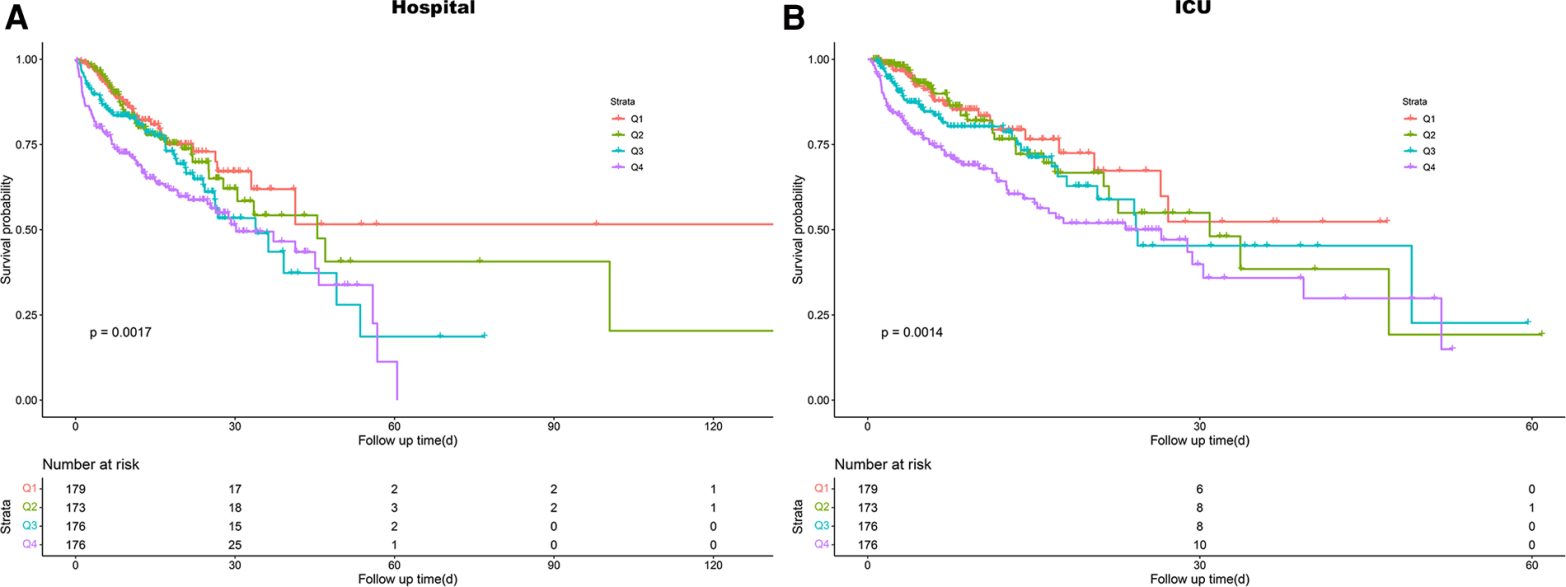
Kaplan–meier survival analysis curves for all-cause mortality. (**A**) Survival probability of all-cause mortality in hospital. (**B**) Survival probability of all-cause mortality in ICU. ICU, intensive care unit.

We plotted Kaplan-Meier survival analysis curves to observe the incidence of the primary endpoint event between groups according to LAR quartiles, as shown in Figure 2. In the hospital, a statistically significant difference in mortality was observed between the groups (log-rank *P* = 0.0017, Figure 2A). Significance was also observed in the ICU (log-rank *P* = 0.0014, Figure 2B).

**Figure 3:**
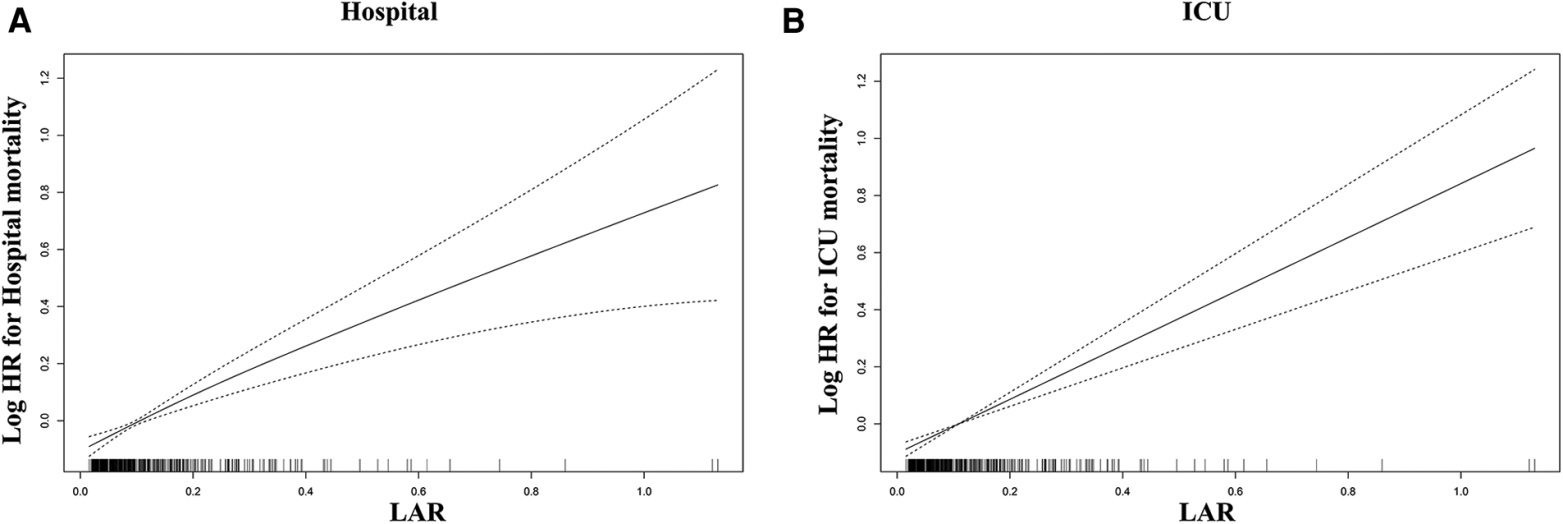
Generalized additive model for association between the LAR and in-hospital mortality. (**A**) Generalized additive model for hospital mortality. (**B**) Generalized additive model for ICU mortality. HR, hazard ratio; ICU, intensive care unit; LAR, lactate to albumin ratio.

**Figure 4:**
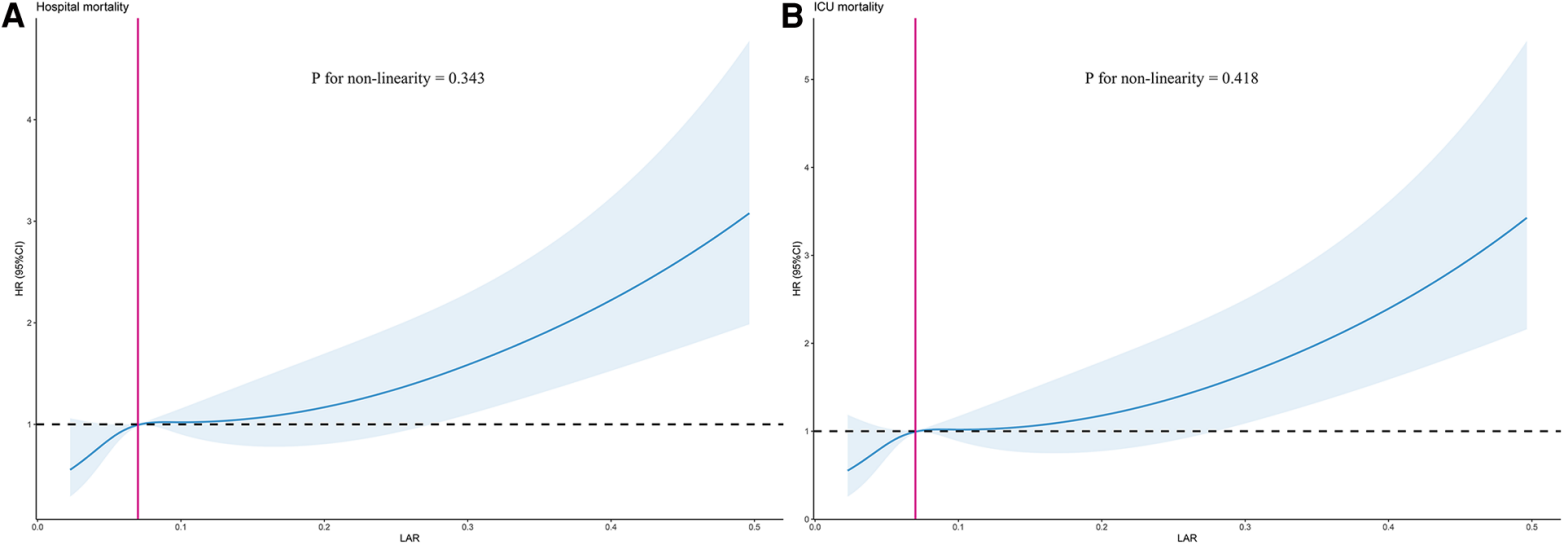
Restricted cubic spline model for association between the LAR and hazard ratio. (**A**) Restricted cubic spline model for hospital mortality. (**B**) Restricted cubic spline model for ICU mortality. HR, hazard ratio; ICU, intensive care unit; LAR, lactate to albumin ratio.

**Figure 5:**
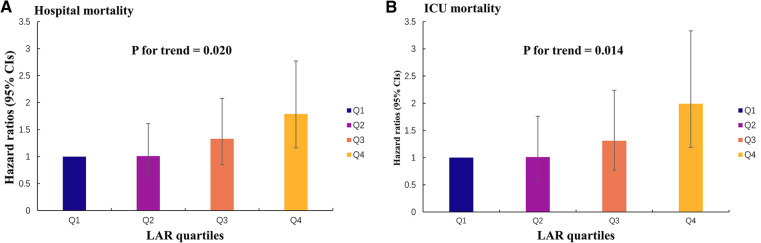
Hazard ratios (95% CIs) for hospital mortality and ICU mortality according to the LAR quartiles after adjusting for model 4. Error bars indicate 95% CIs. The first quartile is the reference. (**A**) Hazard ratios (95% CIs) for hospital mortality. (**B**) Hazard ratios (95% CIs) for ICU mortality. CI, confidence interval; LAR, lactate to albumin ratio.

Using GAM, we found that the relationship between LAR level and in-hospital mortality, including hospital mortality and ICU mortality, was linear (Figure 3). Similar results were seen in the RCS model (*P* for non-linearity = 0.343 and *P* for non-linearity = 0.418, respectively) (Figure 4). We used Cox proportional risk analysis, with the first quartile of the LAR as the reference group, to determine the association between the LAR and in-hospital mortality. The results demonstrated that it was associated with hospital mortality in both model 1 (Q1 vs. Q2: HR, 1.17 [95% CI: 0.75–1.83] *P* = 0.487; Q3: HR, 1.53 [95% CI: 1.00–2.34] *P* = 0.049; Q4: HR, 2.00 [95% CI: 1.34–3.00] *P* < 0.001; *P* for trend = 0.002) and model 4 (Q1 vs. Q2: HR, 1.01 [95% CI: 0.64–1.61] *P* = 0.967; Q3: HR, 1.33 [95% CI: 0.85–2.08] *P* = 0.210; Q4: HR, 1.79 [95% CI: 1.16–2.77] *P* = 0.009; *P* for trend = 0.020), and showed a tendency to increase with the LAR (Table 3; Figure 5A). Further, Cox proportional risk analysis of the LAR and ICU mortality showed similar results (Table 3; Figure 5B).

**Table 3:** Cox proportional hazard ratios (HR) for all-cause mortality.

Categories	Model 1 HR (95% CI)	*P*-value	*P* for trend	Model 2 HR (95% CI)	*P*-value	*P* for trend	Model 3 HR (95% CI)	*P*-value	*P* for trend	Model 4 HR (95% CI)	*P*-value	*P* for trend
Hospital mortality												
Continuous variable	1.34 (1.22–1.47)	< 0.001		1.39 (1.27–1.54)	< 0.001		1.37 (1.24–1.53)	< 0.001		1.39 (1.24–1.56)	< 0.001	
Quartile			0.002			< 0.001			0.013			0.020
Q1 (*n* = 179)	Ref.			Ref.			Ref.			Ref.		
Q2 (*n* = 173)	1.17 (0.75–1.83)	0.487		1.17 (0.75–1.82)	0.499		1.19 (0.76–1.88)	0.447		1.01 (0.64–1.61)	0.967	
Q3 (*n* = 176)	1.53 (1.00–2.34)	0.049		1.47 (0.96–2.26)	0.073		1.44 (0.94–2.22)	0.097		1.33 (0.85–2.08)	0.210	
Q4 (*n* = 176)	2.00 (1.34–3.00)	< 0.001		2.18 (1.46–3.27)	< 0.001		1.89 (1.25–2.87)	0.003		1.79 (1.16–2.77)	0.009	
ICU mortality												
Continuous variable	1.37 (1.24–1.52)	< 0.001		1.42 (1.28–1.57)	< 0.001		1.44 (1.28–1.61)	< 0.001		1.46 (1.29–1.65)	< 0.001	
Quartile			0.002			< 0.001			0.006			0.014
Q1 (*n* = 179)	Ref.			Ref.			Ref.			Ref.		
Q2 (*n* = 173)	1.18 (0.69–2.02)	0.538		1.19 (0.70–2.04)	0.513		1.17 (0.68–2.02)	0.565		1.01 (0.58–1.76)	0.973	
Q3 (*n* = 176)	1.49 (0.89–2.47)	0.126		1.45 (0.87–2.42)	0.157		1.40 (0.84–2.34)	0.201		1.31 (0.77–2.24)	0.324	
Q4 (*n* = 176)	2.21 (1.38–3.53)	< 0.001		2.31 (1.44–3.71)	<0.001		2.13 (1.31–3.46)	0.002		1.99 (1.19–3.33)	0.009	

HR, hazard ratio; CI, confidence interval; Ref., reference

Model 1: unadjusted

Model 2: adjusted for age, gender, and weight

Model 3: adjusted for adjusted for age, gender, weight, CAD, heart failure, atrial fibrillation, hypertension, diabetes, hyperlipidaemia, CKD, stroke, vasopressin use, ventilator use, and RRT

Model 4: adjusted for age, gender, weight, CAD, heart failure, atrial fibrillation, hypertension, diabetes, hyperlipidaemia, CKD, stroke, vasopressin use, ventilator use, RRT, WBC, neutrophil percentage, lymphocyte percentage, platelets, neutrophil/lymphocyte ratio (NLR), platelet/neutrophil ratio (PNR), platelet/lymphocyte ratio (PLR), hemoglobin, potassium, sodium, Scr, BUN, CK-MB, ALT, AST, and SIRS score

### 3.3. Subgroup analysis

**Figure 6:**
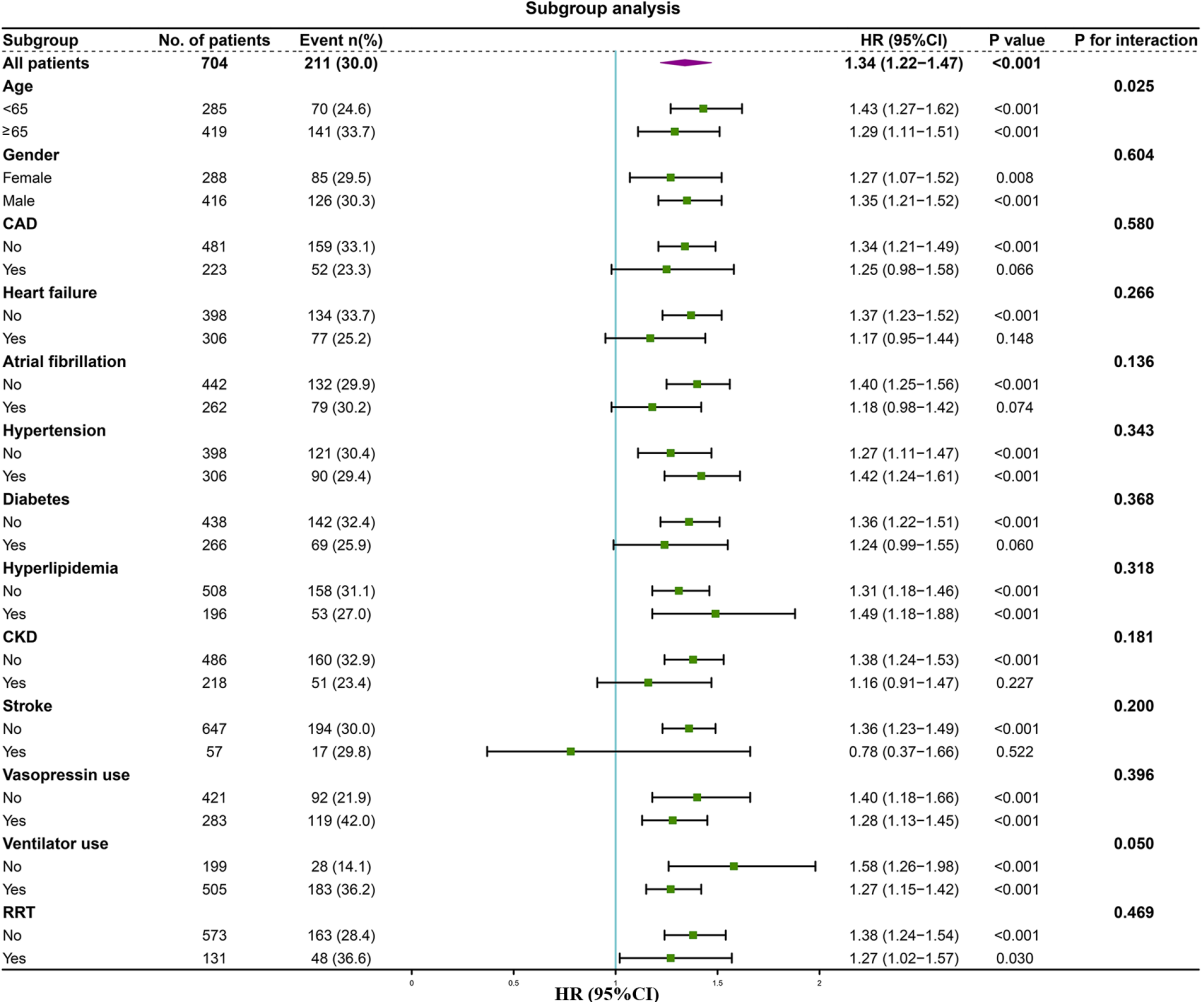
Forest plot of the relationship between hospital mortality and LAR for subgroup analysis. CAD, coronary artery disease; CKD, chronic kidney disease; RRT, renal replacement therapy.

We performed subgroup analysis to assess the association between the LAR and hospital mortality, including age, gender, CAD, heart failure, atrial fibrillation, hypertension, diabetes, hyperlipidemia, CKD, stroke, vasopressin use, ventilator use, and RRT (Figure 6). In the subgroups of age <65 years [HR (95% CI) 1.43 (1.27–1.62)], those age ≥65 years [HR (95% CI) 1.29 (1.11–1.51)], those with female [HR (95% CI) 1.27 (1.07–1.52)], those with male [HR (95% CI) 1.35 (1.21–1.52)], those without CAD [HR (95% CI) 1.34 (1.21–1.49)], those without heart failure [HR (95% CI) 1.37 (1.23–1.52)], those without atrial fibrillation [HR (95% CI) 1.40 (1.25–1.56)], those without hypertension [HR (95% CI) 1.27 (1.11–1.47)], those with hypertension [HR (95% CI) 1.42 (1.24–1.61)], those without diabetes [HR (95% CI) 1.36 (1.22–1.51)], those without hyperlipidemia [HR (95% CI) 1.31 (1.18–1.46)], those with hyperlipidemia [HR (95% CI) 1.49 (1.18–1.88)], those without CKD [HR (95% CI) 1.38 (1.24–1.53)], those without stroke [HR (95% CI) 1.36 (1.23–1.49)], those without vasopressin use [HR (95% CI) 1.40 (1.18–1.66)], those with vasopressin use [HR (95% CI) 1.28 (1.13–1.45)], those without ventilator use [HR (95% CI) 1.58 (1.26–1.98)], those with ventilator use [HR (95% CI) 1.27 (1.15–1.42)], those without RRT [HR (95% CI) 1.38 (1.24–1.54)], and those with RRT [HR (95% CI) 1.27 (1.02–1.57)], the LAR was significantly associated with higher risk of hospital mortality (*P* < 0.05). However, the forest plot showed no significant interaction of LAR by subgroup except for age (*P* for interaction: 0.050–0.604). This indicates that LAR is an independent prognostic factor. Stratified analyses of LAR and ICU mortality showed similar results (Supplementary Figure S1).

### 3.4. ROC curve analysis and prediction of mortality

**Figure 7:**
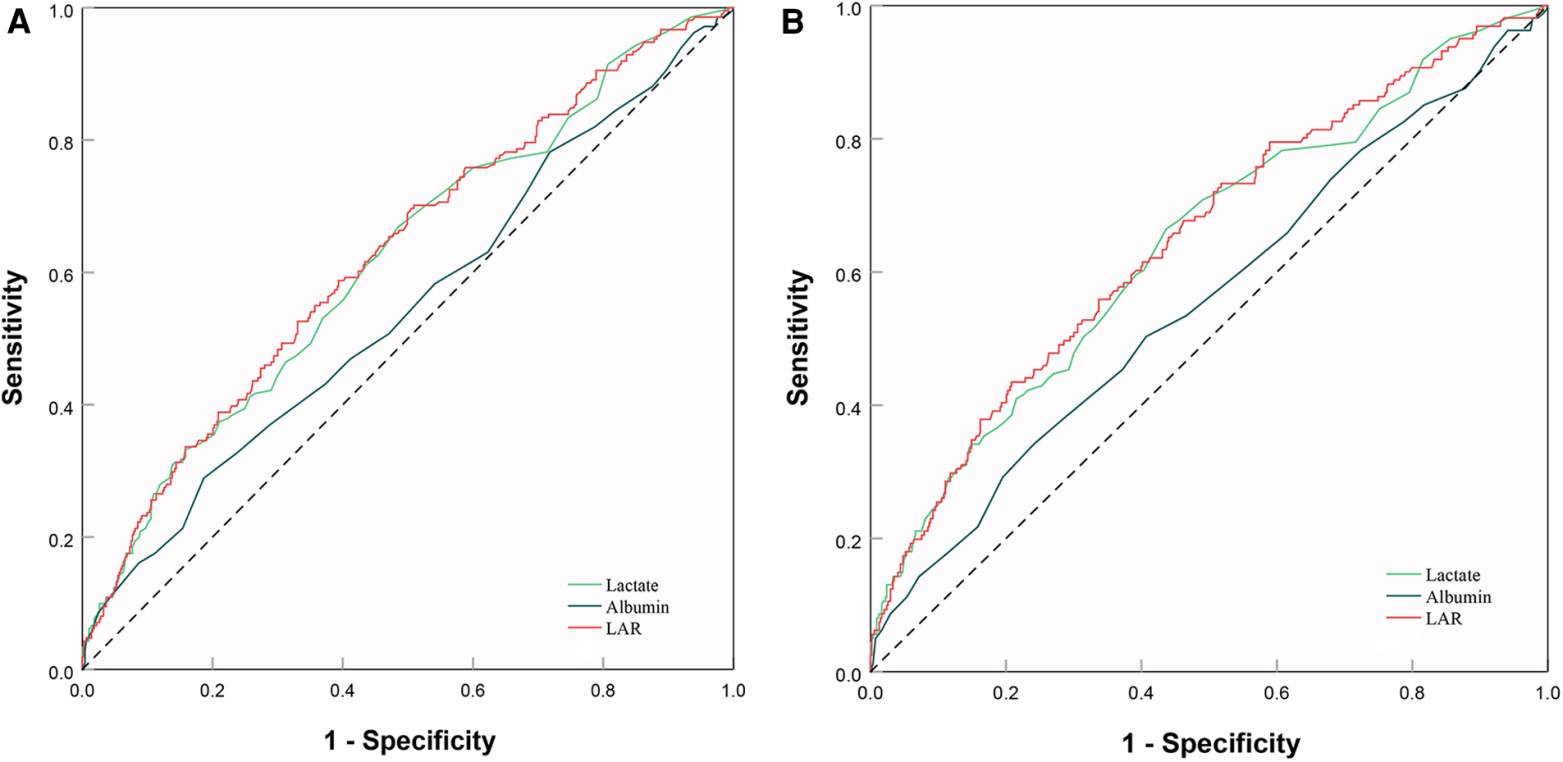
ROC curve analysis and prediction of mortality. (**A**) ROC curves for 3 indicators, LAR, lactate and albumin, to predict hospital mortality. (**B**) ROC curves for 3 indicators, LAR, lactate and albumin, to predict ICU mortality. ROC, receiver operating characteristic; LAR, lactate to albumin ratio.

Firstly, we plotted ROC curves for 3 indicators, LAR, lactate and albumin, to predict in-hospital all-cause mortality in patients with SMI. The result showed that the area under the curve (AUC) of LAR [0.63 (95% CI: 0.58–0.67)] was superior to that of lactate [0.62 (95% CI: 0.57–0.67)] and albumin [0.55 (95% CI: 0.50–0.59)] (Figure 7A). Similar results were found in the ICU mortality (Figure 7B). Thus, LAR had a higher predictive value than lactate and albumin.

Secondly, we also used ROC curves to assess the predictive power of the LAR, SOFA and SAPS II score for in-hospital all-cause mortality in patients with SMI. The AUC of SOFA is 0.62, while the AUC of LAR plus SAPS II is 0.64 for hospital mortality (Supplementary Figure S2A). Furthermore, the AUC of SAPS II plus LAR for ICU mortality rate were 0.66 and 0.67, respectively (Supplementary Figure S2B). When LAR was added to SOFA, IDI showed significant improvements of 2.03% (95% CI: 0.77%–3.30%, *P* = 0.002) and 2.63% (95% CI: 0.96%–4.29%, *P* = 0.002) in both the hospital mortality and ICU mortality. NRI also exhibited statistical significance for both hospital mortality (31.10%, 95% CI: 15.25–46.95%, *P* < 0.001) and ICU mortality (31.61%, 95% CI: 14.23–48.99%, *P* < 0.001) (Table 4).

**Table 4:** The addition of LAR improved the predictive ability of the scoring system for mortality.

Index	AUC (95% CI)	IDI (%) (95% CI)	*P*-value	NRI (%) (95% CI)	*P*-value
Hospital mortality					
LAR	0.63 (0.58–0.67)	–	–	–	–
SOFA	0.62 (0.57–0.67)	Ref.	Ref.	Ref.	Ref.
SAPSⅡ	0.68 (0.64–0.73)	Ref.	Ref.	Ref.	Ref.
LAR + SOFA	0.64 (0.59–0.68)	2.03 (0.77–3.30)	0.002	31.10 (15.25–46.95)	<0.001
LAR + SAPSⅡ	0.70 (0.66–0.74)	2.79 (1.14–4.45)	< 0.001	19.84 (4.69–34.99)	0.010
ICU mortality					
LAR	0.65 (0.60–0.70)	–	–	–	–
SOFA	0.66 (0.61–0.71)	Ref.	Ref.	Ref.	Ref.
SAPSⅡ	0.68 (0.63–0.73)	Ref.	Ref.	Ref.	Ref.
LAR + SOFA	0.67 (0.62–0.72)	2.63 (0.96–4.29)	0.002	31.61 (14.23–48.99)	<0.001
LAR + SAPSⅡ	0.71 (0.66–0.75)	3.90 (1.85–5.95)	< 0.001	29.69 (12.79–46.58)	<0.001

AUC, area under the curve; CI, confidence interval; IDI, integrated discrimination improvement; NRI, net reclassification improvement index; LAR, lactate to albumin ratio; SOFA, Sequential Organ Failure Assessment; SAPS II, Simplifed acute physiological score II; Ref., reference.

The AUC for SAPS II was 0.68, compared with 0.70 for LAR plus SAPS II for hospital mortality (*P* = 0.049) (Supplementary Figure S2C). In addition, for ICU mortality, the AUC for SAPS II and the LAR plus SAPS II were 0.68 and 0.71, respectively (*P* = 0.030) (Supplementary Figure S2D). Similarly, when LAR was added to SAPS II, the IDI and NRI also show significant improvement (Table 4).

## 4. Discussion

SMI is a reversible myocardial injury that requires early assessment to accurately stratify the disease and apply appropriate therapeutic measures. Our research suggests that the LAR can serve as a predictive indicator of mortality in SMI patients.

Serum lactate is a marker of tissue hypoperfusion and is commonly used to evaluate the severity of patients in the ICU (24). Recent study has demonstrated a correlation between lactate levels and adverse outcomes in critically ill patients (25). Furthermore, research has indicated an association between elevated lactate levels and a poor prognosis in sepsis patients (26). Our study also observed elevated serum lactate levels in SMI patients with a poor prognosis. Therefore, elevated lactate levels can be used as an indicator of a poor prognosis in SMI patients. However, it is important to exercise caution when interpreting lactate levels, as certain medications or conditions can also lead to their elevation (27, 28). Consequently, it would be insufficient to rely solely on lactate levels to predict the prognosis of patients with SMI.

Albumin, a multifunctional protein with roles in antioxidant defense, anti-inflammatory action, and maintenance of vascular endothelial function, has been shown to mitigate the detrimental effects of infection-driven inflammatory responses on the body and reduce the incidence of organ failure. However, in patients suffering from severe infections, albumin is markedly reduced, which is associated with adverse clinical outcomes. A plethora of clinical studies have demonstrated that low levels of albumin are linked to increased mortality risk in conditions including sepsis, septic shock, and heart failure (29, 30). Notably, inflammation can augment capillary permeability, leading to leakage of albumin out of blood vessels, resulting in a decline of serum albumin levels (31). Prolonged inflammation may exacerbate this process, ultimately culminating in more severe inflammation (8, 32). In patients with SMI, excessive activation of the immune system leads to extensive albumin consumption, which significantly contributes to poor prognosis.

However, the utility of serum albumin as a prognostic indicator may be limited by other factors such as chronic disease, malnutrition, and inflammation (17). Cakir et al. noted the albumin's prognostic value may be restricted, especially in elderly individuals who are frail (33). Moreover, Finfer et al. showed no significant improvement in mortality among patients with severe sepsis treated with albumin therapy (34). Additionally, Chen et al. indicated that the infusion of exogenous albumin may interfere with the diagnostic capabilities of serum albumin (35). Hence, the prognostic value of albumin is influenced by multiple factors, which limit its clinical utility.

The LAR is a composite indicator that considers both the nutritional and inflammatory status of critically ill patients, thereby reducing the impact of individual factors on regulatory mechanisms. This feature makes it an accurate prognostic biomarker for various diseases. Several studies have demonstrated that LAR has superior predictive value compared to lactate alone in determining outcomes in severely infected patients (19, 33, 36). In patients with severe sepsis and septic shock, elevated LAR levels are associated with the development of multiple organ dysfunction syndrome (MODS) and mortality (37, 38). Notably, Michael et al. have proposed the use of LAR as a novel predictor for stratifying sepsis patients based on the severity of their disease (39). Additionally, LAR has shown promising results in predicting prognosis in critically ill patients with cardiovascular diseases such as heart failure, myocardial infarction, and cardiac arrest (20, 21, 40). Consequently, LAR may serve as a more effective clinical predictor for critically ill patients with SMI. Our study, based on database analysis, found that higher LAR levels were associated with a higher risk of mortality in SMI patients, both during hospitalization and ICU admission, which is consistent with previous findings. Furthermore, we observed that LAR had a better predictive value than either lactate or albumin alone in SMI patients.

To date, there are few studies on the association between LAR and critically ill patients with SMI. Zhang et al. found the diagnostic value of LAR for severe pneumonia and myocardial injury (22). However, the prognostic value of LAR was not evaluated in this study. In addition, our study is the first to establish the significance of LAR as a prognostic marker for hospital mortality and ICU mortality among SMI patients. In our study, higher level of LAR, lactate, and lower albumin levels were observed in the non-survival group of SMI patients. Moreover, we observed that both hospital and ICU mortality rates increased with elevating LAR levels. Similarly, Gharipour et al. found a positive association between LAR and future adverse events in critically ill patients (41). Additionally, another study investigating 1,381 sepsis patients demonstrated that higher LAR was associated with a greater risk of mortality (42). Using Cox proportional risk analysis and Kaplan-Meier survival analysis, we obtained comparable findings: increasing LAR levels correlated positively with an elevated risk of mortality, with the risk gradually escalating as LAR levels increased in SMI patients. Drăgoescu AN et al. showed the predictive potential of NLR for sepsis mortality (43), while Kriplani A et al. found the diagnostic value of NLR and PLR for sepsis (44). In our study, we found that increased LAR was a strong independent predictor of higher mortality in patients with SMI. This finding persisted even after making adjustments for potential confounders such as NLR, PNR, and PLR (Model 4, *P* < 0.001). Furthermore, both the results of the GAM and the RCS model indicated a significant linear correlation between the LAR and all-cause mortality in SMI patients. Importantly, our study established that higher LAR levels correlated with increased hospital and ICU mortality risks. Consequently, our findings permit early identification of patients with high mortality risks, and monitoring LAR may aid in improved management of SMI patients in clinical practice. This is particularly vital for better clinical management to reduce future adverse events.

Our subgroup analysis demonstrated that LAR maintains consistent predictive value across different age and gender groups in patients. Remarkably, Guo W et al. revealed that elevated LAR levels correlated with increased risks of both short- and long-term mortality in patients with heart failure (20). Nevertheless, our study identified a higher risk of mortality in patients without underlying conditions such as CAD, heart failure, atrial fibrillation, CKD, and stroke. Interestingly, as the LAR levels increased, the incidence of these aforementioned diseases decreased, which can be attributed to reverse causality wherein patients with pre-existing conditions were treated more intensively and had their abnormal LAR values corrected more promptly. Nonetheless, our forest plots indicated no significant interaction (*P* for interaction >0.05) between LAR and these subgroups, suggesting that the presence or absence of these diseases did not affect the predictive power of LAR.

The findings from our study demonstrate that LAR is a superior predictor of mortality in patients suffering from SMI. The results of our ROC analysis revealed an AUC of 0.63 for LAR in the hospital and 0.65 in the ICU. These values were higher than those observed for lactate, which were 0.62 and 0.64 respectively. Moreover, these results were consistent with prior research (18). The optimal cutoff value for predicting mortality was determined to be 0.09 for LAR (positive predictive value (PPV): 41%, negative predictive value (NPV): 77%) and 0.12 for ICU settings (PPV: 38%, NPV: 83%). A study conducted by Lichtenauer et al. on 348 sepsis patients identified an optimal cutoff value of 0.15, which was slightly greater than our findings (39). However, the discrepancy could be attributed to differences in sample size, and we recommend conducting a future prospective study to determine the optimal LAR value. Importantly, when we add LAR to SOFA and SAPS II, the IDI and NRI showed significant improvement, both in hospital mortality and ICU mortality. The above results indicate that LAR may improve the ability of severity scores to predict mortality risk.

The primary strength of our study is its confirmation of the strong association between increased LAR and higher mortality rates in SMI patients. Additionally, LAR is a simple and easily obtainable indicator when compared to other indicators. Nonetheless, several limitations should be noted. Firstly, the study was a single-center retrospective study, and therefore, causality cannot be established. Secondly, due to the large number of missing variables in the database, unadjusted confounders may have affected our results despite multivariate adjustment and subgroup analysis. Finally, our study only examined the relationship between LAR at admission and prognosis and did not assess the impact of changes in LAR on prognosis. Therefore, further studies are necessary to determine if dynamic changes in LAR can also predict mortality.

## 5. Conclusions

According to our research findings, there exists a positive correlation between LAR and the mortality rate of patients in ICU and hospitals who suffer from SMI. The risk of mortality in SMI patients increases proportionally with higher LAR values. Our study posits that LAR could potentially serve as a predictive indicator for mortality in SMI patients, contributing to the stratification of risk levels and prognosis prediction. It is recommended that further prospective studies be conducted to validate the predictive efficacy of LAR in SMI patients.

## Data Availability

Publicly available datasets were analyzed in this study. This data can be found here: https://mimic.mit.edu.
