# Supplementary material for: The lactate to albumin ratio linked to all-cause mortality in critically ill patients with septic myocardial injury
Source: Front Cardiovasc Med. 2023 Sep 13;10:1233147. doi: 10.3389/fcvm.2023.1233147 (PMC10542581; doi:10.3389/fcvm.2023.1233147)
Supplement: Supplementary file 1 [file Datasheet1.docx]

Supplementary Material

The lactate to albumin ratio linked to all-cause mortality in critically ill patients with septic myocardial injury

**Sheng Chen, Senhong Guan, Zhaohan Yan, Fengshan Ouyang, Shuhuan Li, Lanyuan Liu,** **Jiankai Zhong^*^**

*** Correspondence:** Jiankai Zhong: doctor-zh@smu.edu.cn

# Supplementary Figures and Tables

## Supplementary Figures


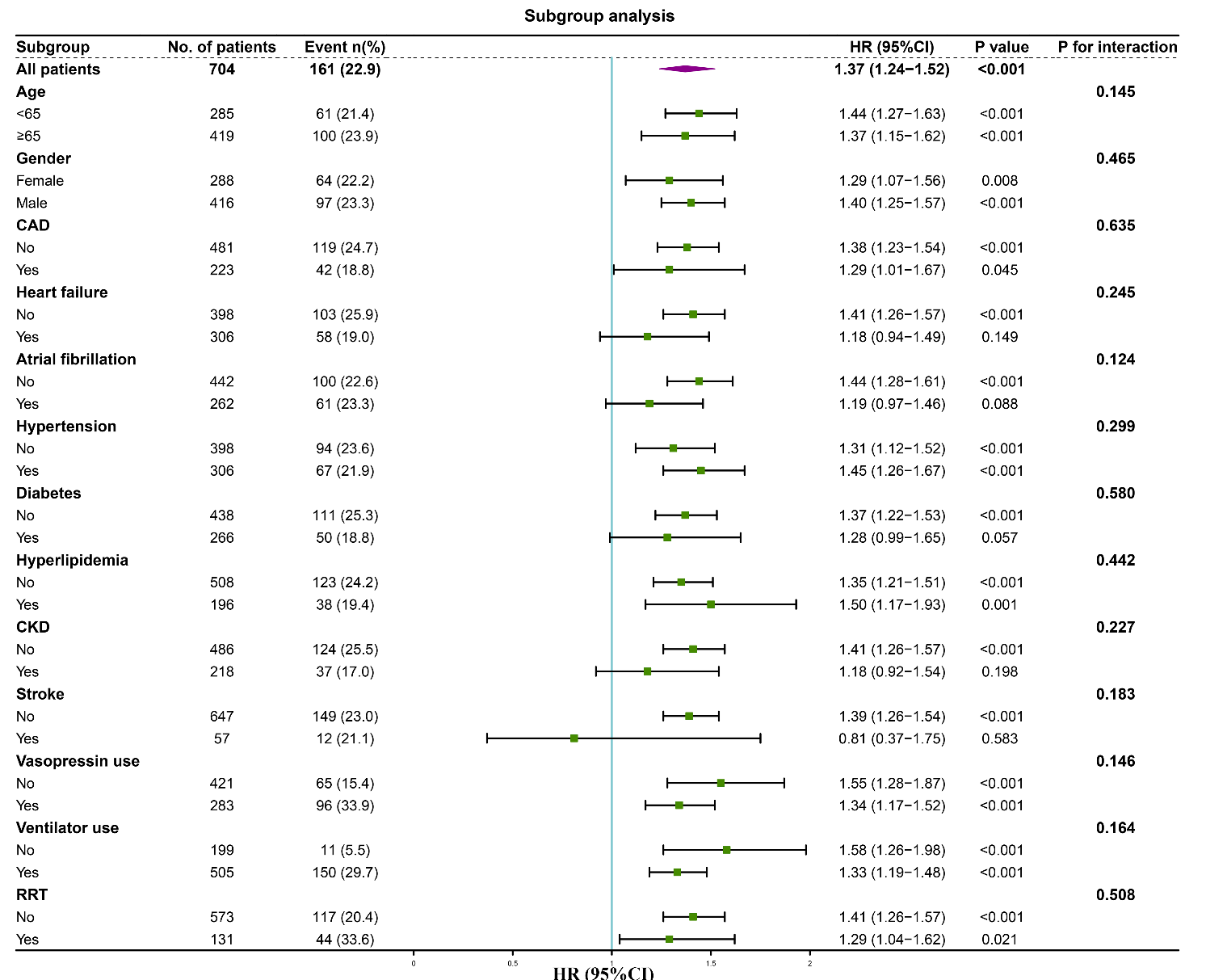


**Supplementary Figure 1.** Forest plot of the relationship between ICU mortality and LAR for subgroup analysis. CAD, coronary artery disease; CKD, chronic kidney disease; RRT, renal replacement therapy


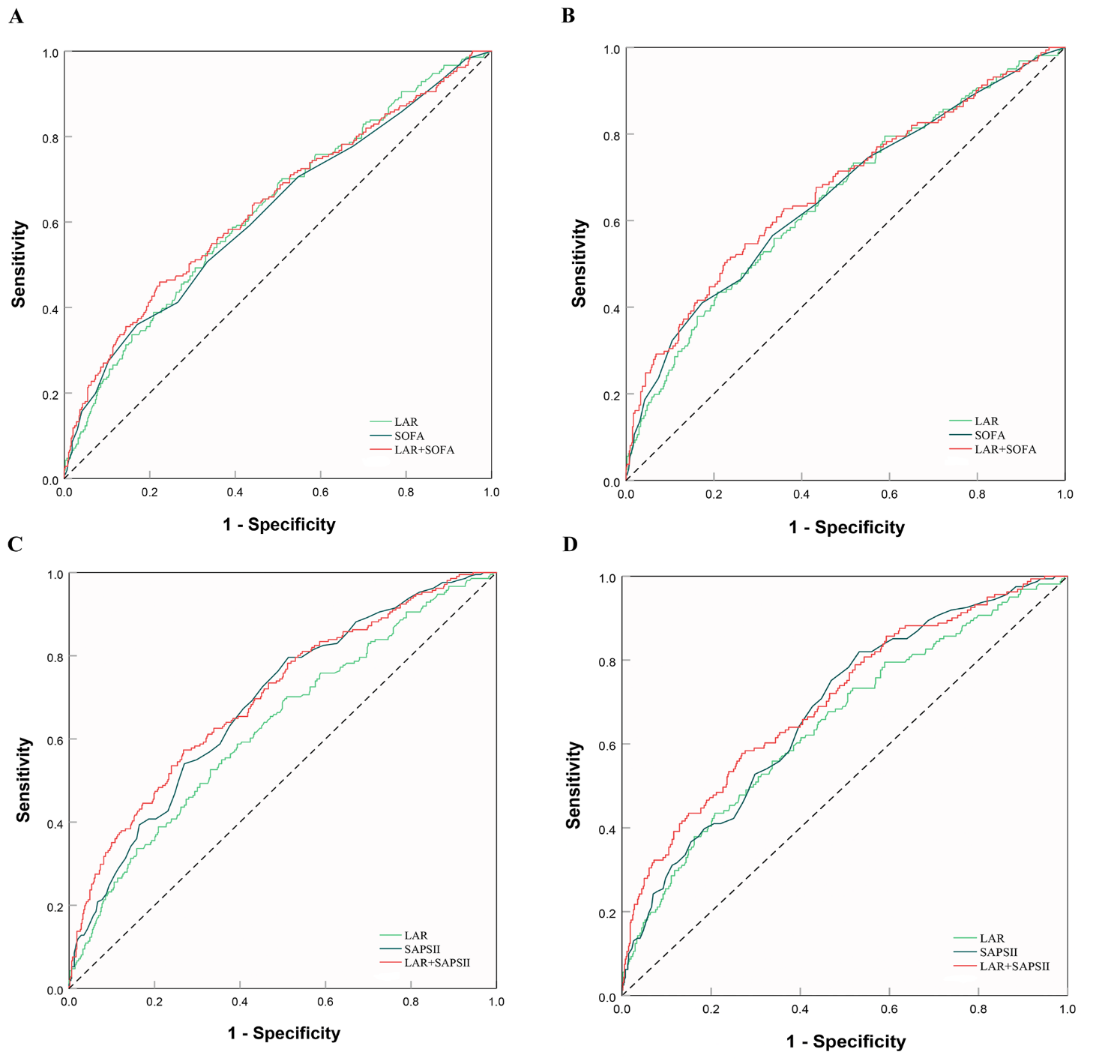


**Supplementary Figure 2.** ROC curve analysis and prediction of mortality. (A) ROC curves for LAR and SOFA score to predict hospital mortality. (B) ROC curves for LAR and SOFA score to predict ICU mortality. (C) ROC curves for LAR and SAPSⅡ score to predict hospital mortality. (D) ROC curves for LAR and SAPSⅡ score to predict ICU mortality. Abbreviations: ROC, receiver operating characteristic; LAR, lactate to albumin ratio; SOFA, Sequential Organ Failure Assessment; SAPSⅡ, Simplifed acute physiological score Ⅱ

## Supplementary Table

**Supplementary Table 1. Missing number for variables**

| **Variables** | Missing number (%) |
| --- | --- |
| Age | 0 (0) |
| Gender | 0 (0) |
| Weight | 3 (00.42%) |
| Height | 240 (34.10%) |
| CAD | 0 (0) |
| Heart failure | 0 (0) |
| Atrial fibrillation | 0 (0) |
| Hypertension | 0 (0) |
| Diabetes | 0 (0) |
| Hyperlipidemia | 0 (0) |
| CKD | 0 (0) |
| Stoke | 0 (0) |
| WBC | 0 (0) |
| Neutrophil percentage | 0 (0) |
| Lymphocyte percentage | 0 (0) |
| Platelet | 0 (0) |
| NLR | 0 (0) |
| PNR | 0 (0) |
| PLR | 0 (0) |
| Hemoglobin | 0 (0) |
| Hematocrit | 0 (0) |
| Potassium | 1 (00.14%) |
| Sodium | 1 (00.14%) |
| Albumin | 0 (0) |
| Lactate | 0 (0) |
| Scr | 7 (1.00%) |
| BUN | 0 (0) |
| cTNT | 0 (0) |
| CK-MB | 118 (16.76%) |
| ALT | 68 (9.66%) |
| AST | 68 (9.66%) |
| LDH | 191 (27.13%) |
| LAR | 0 (0) |
| SOFA score | 0 (0) |
| SIRS score | 0 (0) |
| qSOFA score | 0 (0) |
| APSⅢ | 0 (0) |
| SAPSⅡ | 0 (0) |
| Vasopressin use | 0 (0) |
| Ventilator use | 39 (5.53%) |
| RRT | 0 (0) |

CAD, coronary artery disease; CKD, chronic kidney disease; WBC, white blood cell; NLR, neutrophil/lymphocyte ratio; PNR, platelet/neutrophil ratio; PLR, platelet/lymphocyte ratio; Scr, serum creatinine; BUN, blood urea nitrogen; cTNT, cardiac troponin T; CK-MB, creatine kinase MB isoenzyme; ALT, alanine aminotransferase; AST, aspartate aminotransferase; LDH, lactate dehydrogenase; LAR, lactate to albumin ratio; SOFA, Sequential Organ Failure Assessment; SIRS, Systemic inflammatory response syndrome; qSOFA, quick Sequential Organ Failure Assessment; APSⅢ, Acute physiology score Ⅲ; SAPSⅡ, Simplifed acute physiological score Ⅱ; RRT, renal replacement therapy.

**Supplementary Table 2. Baseline characteristics of the Survivor and Non-survivor groups.**

| **Characteristics** | **Overall (n=704)** | **Survivors (n=493)** | **Non-survivors (n=211)** | ***P*-value** |
| --- | --- | --- | --- | --- |
| Age, years | 68.94 (57.78, 80.55) | 67.82 (57.18, 79.46) | 72.88 (60.12, 82.01) | 0.015 |
| Gender, n (%) |  |  |  | 0.825 |
| Female | 288 (40.90) | 203 (41.18) | 85 (40.28) |  |
| Male | 416 (59.10) | 290 (58.82) | 126 (59.72) |  |
| Weight, Kg | 80.00 (67.00, 96.67) | 80.30 (67.05, 95.00) | 80.00 (66.70, 99.50) | 0.912 |
| Comorbidities, n (%) |  |  |  |  |
| CAD | 223 (31.70) | 171 (34.68) | 52 (24.64) | 0.009 |
| Heart failure | 306 (43.50) | 229 (46.45) | 77 (36.49) | 0.015 |
| Atrial fibrillation | 262 (37.22) | 183 (37.12) | 79 (37.44) | 0.936 |
| Hypertension | 306 (43.50) | 216 (43.81) | 90 (42.65) | 0.776 |
| Diabetes | 266 (37.80) | 197 (39.96) | 69 (32.70) | 0.069 |
| Hyperlipidemia | 196 (27.84) | 143 (29.01) | 53 (25.12) | 0.292 |
| CKD | 218 (30.96) | 167 (33.87) | 51 (24.17) | 0.010 |
| Stroke | 57 (8.09) | 40 (8.11) | 17 (8.06) | 0.980 |
| Laboratory parameters |  |  |  |  |
| WBC, K/uL | 12.70 (8.60, 17.60) | 12.30 (8.45, 17.25) | 13.50 (8.70, 18.20) | 0.155 |
| Neutrophil, % | 83.95 (76.00, 88.97) | 83.50 (76.00, 88.75) | 84.70 (74.60, 89.50) | 0.424 |
| Lymphocyte, % | 8.30 (5.10, 13.00) | 8.70 (5.35, 13.40) | 7.40 (4.80, 11.90) | 0.028 |
| Platelet, K/uL | 203.00 (140.25, 274.00) | 207.00 (148.50, 277.00) | 198.00 (116.00, 271.00) | 0.019 |
| NLR | 9.94 (5.98, 16.65) | 9.35 (5.86, 16.38) | 10.75 (6.40, 18.48) | 0.098 |
| PNR | 20.57 (12.91, 31.21) | 21.95 (13.96, 32.28) | 17.24 (11.77, 28.26) | 0.005 |
| PLR | 195.16 (119.89, 337.87) | 193.59 (120.59, 335.70) | 198.23 (112.76, 344.95) | 0.797 |
| Hemoglobin, g/dL | 10.70 (9.20, 12.30) | 10.60 (9.15, 12.30) | 10.70 (9.30, 12.40) | 0.410 |
| Hematocrit, % | 32.20 (27.80, 37.00) | 32.00 (27.60, 36.40) | 32.70 (28.10, 38.40) | 0.105 |
| Potassium, mEq/L | 4.20 (3.70, 4.70) | 4.10 (3.70, 4.70) | 4.40 (3.80, 4.90) | 0.006 |
| Sodium, mEq/L | 139.00 (136.00, 142.00) | 139.00 (136.00, 142.00) | 139.00 (136.00, 143.00) | 0.918 |
| Lactate, mmol/L | 2.10 (1.40, 3.60) | 1.90 (1.30, 3.23) | 2.50 (1.70, 4.70) | < 0.001 |
| Albumin, g/dL | 3.00 (2.50, 3.40) | 3.00 (2.60, 3.40) | 2.90 (2.40, 3.30) | 0.049 |
| Scr, mg/dL | 1.50 (1.00, 2.30) | 1.40 (1.00, 2.30) | 1.60 (1.10, 2.30) | 0.056 |
| BUN, mg/dL | 31.00 (20.00, 50.75) | 30.00 (19.00, 49.00) | 34.00 (23.00, 53.00) | 0.009 |
| cTNT, ng/ml | 0.11 (0.04, 0.36) | 0.10 (0.04, 0.31) | 0.12 (0.05, 0.49) | 0.300 |
| CK-MB, ng/mL | 9.00 (5.00, 22.00) | 9.00 (5.00, 20.45) | 11.00 (6.00, 26.00) | 0.031 |
| ALT, IU/L | 47.00 (24.00, 106.00) | 44.00 (22.00, 90.33) | 58.00 (29.00, 157.00) | 0.002 |
| AST, IU/L | 70.28 (37.00, 174.75) | 63.00 (63.00, 138.00) | 99.00 (51.00, 291.00) | < 0.001 |
| LAR | 0.07 (0.05, 0.12) | 0.07 (0.04, 0.11) | 0.09 (0.06, 0.18) | < 0.001 |
| Scoring systems |  |  |  |  |
| SOFA score | 7.00 (5.00, 10.00) | 7.00 (5.00, 10.00) | 9.00 (6.00, 12.00) | < 0.001 |
| SIRS score | 3.00 (3.00, 4.00) | 3.00 (3.00, 4.00) | 3.00 (3.00, 4.00) | 0.148 |
| qSOFA score | 2.00 (2.00, 3.00) | 2.00 (2.00, 3.00) | 2.00 (2.00, 3.00) | 0.150 |
| APSⅢ | 64.00 (49.00, 82.00) | 61.00 (46.00, 76.00) | 75.00 (57.00, 96.00) | < 0.001 |
| SAPSⅡ | 49.00 (39.00, 60.00) | 46.00 (37.00, 56.00) | 55.00 (47.00, 66.00) | < 0.001 |
| Clinical treatment, n (%) |  |  |  |  |
| Vasopressin use | 283 (40.19) | 164 (33.26) | 119 (56.39) | < 0.001 |
| Ventilator use | 505 (71.73) | 322 (65.31) | 183 (86.73) | < 0.001 |
| RRT | 131 (18.61) | 83 (16.84) | 48 (22.75) | 0.065 |
| Event |  |  |  |  |
| LOS Hospital, days | 11.90 (6.60, 21.68) | 13.80 (7.99, 22.98) | 7.33 (3.10, 16.91) | < 0.001 |

CAD, coronary artery disease; CKD, chronic kidney disease; WBC, white blood cell; NLR, neutrophil/lymphocyte ratio; PNR, platelet/neutrophil ratio; PLR, platelet/lymphocyte ratio; SCr, serum creatinine; BUN, blood urea nitrogen; cTNT, cardiac troponin T; CK-MB, creatine kinase MB isoenzyme; ALT, alanine aminotransferase; AST, aspartate aminotransferase; LAR, lactate to albumin ratio; SOFA, Sequential Organ Failure Assessment; SIRS, Systemic inflammatory response syndrome; qSOFA, quick Sequential Organ Failure Assessment; APSⅢ, Acute physiology score Ⅲ; SAPSⅡ, Simplifed acute physiological score Ⅱ; RRT, renal replacement therapy; LOS, length of stay.
